# Supplementary material for: Two-step detection of Lewy body pathology via smell-function testing and CSF α-synuclein seed amplification
Source: Nat Commun. 2025 Aug 5;16:7182. doi: 10.1038/s41467-025-62458-7 (PMC12325632; doi:10.1038/s41467-025-62458-7)
Supplement: Supplementary file 1 — Supplementary Information [file 41467_2025_62458_MOESM1_ESM.pdf]

SUPPLEMENTAL RESULTS

Table S1. Participant characteristics at baseline

	**Whole cohort (*N*=358)**
**Age at death, years**	86.2 (7.8)
**Sex, n female**	153 (42.7%)
**UPSIT score**	20.8 (8.6)
**Interval UPSIT and death, years**	3.2 (2.3)
**CSF SAA+, n**	152 (42.5%)
**LBP_{any}+, n**	170 (47.5%)
**LBP_{ctx}+, n**	126 (35.2%)
**$LBP_{B/L-N}$+, n**	107 (29.9%)
**PMI, hours**	3.9 (3.9)

B/L-N = brainstem/limbic or neocortical; CSF = cerebrospinal fluid; ctx = cortex; LBP = Lewy body pathology; PMI = postmortem interval; UPSIT = University of Pennsylvania Smell Identification Test.

Table S2. Clinicopathological diagnosis

	Overall (N=358)
**AD**	68 (19.0%)
**AD and DLB**	25 (7.0%)
**AD, DLB, and VaD**	1 (0.3%)
**AD and HS**	3 (0.8%)
**AD and PD**	15 (4.2%)
**AD, PD, and HS**	1 (0.3%)
**AD, PD, and PSP**	1 (0.3%)
**AD and PSP**	4 (1.1%)
**AD, PSP, and FTLD-TDP**	1 (0.3%)
**AD and VaD**	11 (3.1%)
**AD, VaD, and FTLD-TDP**	1 (0.3%)
**AD, VaD, and PSP**	1 (0.3%)
**Control**	58 (16.2%)
**ILBD**	18 (5.0%)
**MCI NOS**	56 (15.6%)
**PSP**	19 (5.3%)
**MND**	1 (0.3%)
**Dementia NOS**	4 (1.1%)
**HS, MND, and FTLD-TDP**	1 (0.3%)
**MSA**	3 (0.8%)
**PD**	47 (13.1%)
**PD and CBD**	2 (0.6%)
**PD and PSP**	5 (1.4%)
**DLB and HS**	1 (0.3%)
**DLB**	1 (0.3%)
**AD and PSP**	1 (0.3%)
**AD, VaD, PSP, and HS**	1 (0.3%)
**VaD**	4 (1.1%)
**VaD, HS, and FTLD-TDP**	1 (0.3%)
**PICKS disease**	1 (0.3%)
**VaD and CBD**	1 (0.3%)
**MS**	1 (0.3%)

AD = Alzheimer’s disease; CBD = corticobasal degeneration; DLB = dementia with Lewy

bodies; FTLD = frontotemporal lobar degeneration; HS = hippocampal sclerosis; ILBD = incidental Lewy body disease; MCI = mild cognitive impairment; MND = motor neuron disease; MS = multiple sclerosis; MSA = multiple system atrophy; NOS = not otherwhise specified; PD = Parkinson's disease; PSP = progressive supranuclear palsy; VaD = vascular dementia.

Table S3. *Antemortem* clinical diagnosis

	**Overall (N=358)**
**AD**	3 (0.8%)
**AD and Lewy body disease**	1 (0.3%)
**AD and/or CvD**	1 (0.3%)
**AD and DLB**	1 (0.3%)
**AD and VaD**	6 (1.7%)
**ALS**	1 (0.3%)
**Clinically impaired, NOS**	86 (24.0%)
**Clinically impaired, NOS or FTD**	1 (0.3%)
**DLB**	7 (2.0%)
**DLB, AD, or PD**	1 (0.3%)
**DLB or probable AD**	1 (0.3%)
**Cognitively unimpaired**	78 (21.8%)
**Cognitively unimpaired or clinically impaired NOS**	4 (1.1%)
**Cognitively unimpaired, parkinsonism**	5 (1.4%)
**Mixed AD**	1 (0.3%)
**Mixed VaD**	2 (0.6%)
**MSA**	1 (0.3%)
**MSA and NPH**	1 (0.3%)
**PD**	64 (17.9%)
**PD and DLB**	1 (0.3%)
**PD or DLB**	1 (0.3%)
**PD, DLB, FTD, or PSP**	1 (0.3%)
**PD, MSA, PSP, or CBD**	1 (0.3%)
**PLS**	1 (0.3%)
**Possible AD**	18 (5.0%)
**Possible AD and VaD**	1 (0.3%)
**Possible AD or VaD**	1 (0.3%)
**Possible DLB**	1 (0.3%)
**Possible DLB, CBD, or MSA**	1 (0.3%)
**Possible PD**	2 (0.6%)
**PPA due to FTD or AD**	2 (0.6%)
**PPA NOS**	1 (0.3%)
**Probable AD**	44 (12.3%)
**Probable AD and DLB**	2 (0.6%)
**Probable PD**	4 (1.1%)
**PSP**	3 (0.8%)
**VaD**	8 (2.2%)

AD = Alzheimer's disease; ALS = amyotrophic lateral sclerosis; CBD = corticobasal degeneration; CvD = cerebrovascular disease; DLB = dementia with Lewy bodies; FTD = frontotemporal dementia; MSA = multiple system atrophy; NOS = not otherwhise specified; NPH = normal pressure hydrocephalus; PD = Parkinson's disease; PLS = primary lateral scerosis; PPA = primary progressive aphasia; PSP = progressive supranuclear palsy; VaD = vascular dementia.

Table S4. Results of the 5-fold cross-validated logistic regression model predicting *postmortem* cortical LBP

	**St. Estimate**	**Std. error**	**Z value**	***P* value**
Fold 1				
**Intercept**	10.73	1.79	5.99	$2.16*10^{-9}$
**UPSIT score**	-0.22	0.03	-8.70	$<2.00*10^{-16}$
**Age at time of UPSIT**	-0.09	0.02	-4.4	$1.07*10^{-5}$
**Sex (female)**	-0.36	0.32	-1.12	0.263
Fold 2				
**Intercept**	13.20	2.06	6.41	$1.48*10^{-10}$
**UPSIT score**	-0.26	0.29	-8.88	$<2.00*10^{-16}$
**Age at time of UPSIT**	-0.11	0.02	-4.83	$1.35*10^{-6}$
**Sex (female)**	-0.54	0.34	-1.60	0.110
Fold 3				
**Intercept**	11.49	1.85	6.21	$5.40*10^{-10}$
**UPSIT score**	-0.21	0.02	-8.69	$<2.00*10^{-16}$
**Age at time of UPSIT**	-0.10	0.02	-4.80	$1.58*10^{-6}$
**Sex (female)**	0.03	0.32	0.08	0.934
Fold 4				
**Intercept**	10.90	1.88	5.78	$7.37*10^{-9}$
**UPSIT score**	-0.22	0.03	-8.60	$<2.00*10^{-16}$
**Age at time of UPSIT**	-0.09	0.02	-4.33	$1.51*10^{-5}$
**Sex (female)**	-0.28	0.32	-0.89	0.372
Fold 5				
**Intercept**	10.40	1.76	5.91	$3.45*10^{-9}$
**UPSIT score**	-0.21	0.02	-8.73	$<2.00*10^{-16}$
**Age at time of UPSIT**	-0.09	0.02	-4.29	$1.77*10^{-5}$
**Sex (female)**	-0.39	0.32	-1.20	0.230

Two-sided 5-fold cross-validated logistic regression model predicting *postmortem* cortical Lewy body pathology with UPSIT scores, age at time of UPSIT, and sex as predictors. Data correspond to the model across 1000 iterations that yielded median accuracy. Results are shown for each fold seperately. Estimates are standardized. LBP = Lewy body pathology; st = standard; std = standardized; UPSIT = University of Pennsylvania Smell Identification Test.

Table S5. Two-step workflow performance for detecting cortical LB pathology

Approach	Sensitivity	Median accuracy (CI)	Median PPV (CI)	Median NPV (CI)	Median TP (CI)	Median FP (CI)	Median TN (CI)	Median FN (CI)
Whole cohort								
Two-step	80%	0.91 (0.90-0.92)	0.94 (0.93-0.95)	0.89 (0.88-0.90)	99 (96-102)	6 (5-7)	226 (225-227)	27 (24-30)
Two-step	85%	0.92 (0.91-0.93)	0.94 (0.94-0.95)	0.92 (0.90-0.92)	105 (102-107)	7 (6-7)	225 (225-226)	21 (19-24)
Two-step	90%	0.94 (0.93-0.94)	0.93 (0.92-0.94)	0.94 (0.93-0.95)	111 (109-113)	8 (7-9)	224 (223-225)	15 (13-17)
Two-step	95%	0.94 (0.93-0.95)	0.90 (0.89-0.92)	0.96 (0.95-0.97)	118 (115-119)	13 (11-15)	219 (217-221)	8 (7-11)
UPSIT-only	80%	0.81 (0.80-0.82)	0.71 (0.70-0.72)	0.88 (0.87-0.89)	100 (97-103)	41 (39-44)	191 (188-193)	26 (23-29)
UPSIT-only	85%	0.81 (0.80-0.82)	0.69 (0.68-0.71)	0.90 (0.89-0.92)	106 (103-109)	47 (44-51)	185 (181-188)	20 (17-23)
UPSIT-only	90%	0.79 (0.79-0.80)	0.65 (0.64-0.66)	0.93 (0.92-0.94)	113 (111-115)	61 (57-64)	171 (168-175)	13 (11-15)
UPSIT-only	95%	0.74 (0.72-0.76)	0.58 (0.57-0.60)	0.96 (0.94-0.97)	120 (117-121)	86 (80-92)	146 (140-152)	6 (5-9)
CSF-only	-	0.92 (0.89-0.94)	0.82 (0.76-0.88)	0.99 (0.98-1.00)	125 (108-142)	28 (18-38)	204 (186-221)	2 (0-5)
Clinical parkinsonism								
Two-step	80%	0.92 (0.90-0.93)	1.00 (0.99-1.00)	0.82 (0.79-0.85)	82 (79-84)	0 (0-1)	56 (55-56)	12 (10-15)
Two-step	85%	0.93 (0.92-0.95)	0.99 (0.99-1.00)	0.86 (0.83-0.89)	85 (83-87)	1 (0-1)	55 (55-56)	9 (7-11)
Two-step	90%	0.95 (0.94-0.97)	0.99 (0.98-0.99)	0.90 (0.89-0.93)	88 (87-90)	1 (1-2)	55 (54-55)	6 (4-7)
Two-step	95%	0.95 (0.95-0.97)	0.96 (0.95-0.98)	0.95 (0.93-0.96)	91 (90-92)	4 (2-5)	52 (51-54)	3 (2-4)
UPSIT-only	80%	0.86 (0.84-0.87)	0.90 (0.88-0.90)	0.81 (0.77-0.84)	83 (80-85)	9 (9-11)	47 (45-47)	11 (9-14)
UPSIT-only	85%	0.87 (0.85-0.89)	0.89 (0.87-0.90)	0.86 (0.82-0.90)	87 (84-89)	11 (10-13)	45 (43-46)	7 (5-10)
UPSIT-only	90%	0.87 (0.86-0.89)	0.86 (0.84-0.87)	0.91 (0.89-0.95)	90 (89-92)	15 (13-17)	41 (39-43)	4 (2-5)
UPSIT-only	95%	0.85 (0.83-0.87)	0.81 (0.79-0.83)	0.97 (0.94-1.00)	93 (92-94)	22 (19-25)	34 (31-37)	1 (0-2)
CSF-only	-	0.95 (0.92-0.98)	0.95 (0.90-0.99)	0.96 (0.91-1.00)	92 (80-104)	5 (1-9)	51 (40-63)	2 (0-5)
Clinical Alzheimer's disease								
Two-step	80%	0.89 (0.88-0.91)	0.83 (0.82-0.87)	0.91 (0.89-0.93)	19 (18-21)	4 (3-4)	67 (67-68)	7 (5-8)
Two-step	85%	0.91 (0.90-0.93)	0.84 (0.83-0.85)	0.93 (0.92-0.96)	21 (20-23)	4 (4-4)	67 (67-67)	5 (3-6)
Two-step	90%	0.95 (0.92-0.95)	0.86 (0.83-0.86)	0.99 (0.96-0.99)	25 (23-25)	4 (4-5)	67 (66-67)	1 (1-3)

Two-step	95%	0.94 (0.93-0.94)	0.83 (0.81-0.83)	0.99 (0.99-0.99)	25 (25-25)	5 (5-6)	66 (65-66)	1 (1-1)
UPSIT-only	80%	0.71 (0.69-0.73)	0.48 (0.45-0.50)	0.88 (0.86-0.91)	19 (18-21)	21 (19-23)	50 (48-52)	7 (5-8)
UPSIT-only	85%	0.70 (0.67-0.72)	0.47 (0.44-0.49)	0.90 (0.88-0.94)	21 (20-23)	25 (23-26)	46 (45-48)	5 (3-6)
UPSIT-only	90%	0.69 (0.67-0.71)	0.46 (0.44-0.48)	0.98 (0.93-0.98)	25 (23-25)	28 (27-30)	43 (41-44)	1 (1-3)
UPSIT-only	95%	0.60 (0.57-0.63)	0.40 (0.38-0.42)	0.97 (0.97-0.97)	25 (25-25)	38 (35-41)	33 (30-36)	1 (1-1)
CSF-only	-	0.92 (0.86-0.97)	0.76 (0.62-0.90)	1.00 (1.00-1.00)	26 (18-35)	8 (3-14)	65 (53-72)	0 (0-0)
Clinically unimpaired								
Two-step	80%	0.91 (0.91-0.91)	1.00 (1.00-1.00)	0.91 (0.91-0.91)	1 (1-1)	0 (0-0)	39 (39-39)	4 (4-4)
Two-step	85%	0.91 (0.91-0.91)	1.00 (1.00-1.00)	0.91 (0.91-0.91)	1 (1-1)	0 (0-0)	39 (39-39)	4 (4-4)
Two-step	90%	0.89 (0.89-0.91)	0.50 (0.50-1.00)	0.91 (0.91-0.91)	1 (1-1)	1 (0-1)	38 (38-39)	4 (4-4)
Two-step	95%	0.93 (0.91-0.93)	0.75 (0.67-0.75)	0.95 (0.93-0.95)	3 (2-3)	1 (1-1)	38 (38-38)	2 (2-3)
UPSIT-only	80%	0.89 (0.89-0.89)	0.50 (0.50-0.50)	0.91 (0.91-0.91)	1 (1-1)	1 (1-1)	38 (38-38)	4 (4-4)
UPSIT-only	85%	0.89 (0.84-0.89)	0.50 (0.25-0.50)	0.91 (0.90-0.91)	1 (1-1)	1 (1-3)	38 (36-38)	4 (4-4)
UPSIT-only	90%	0.82 (0.80-0.86)	0.20 (0.17-0.33)	0.90 (0.90-0.90)	1 (1-1)	4 (2-5)	35 (34-37)	4 (4-4)
UPSIT-only	95%	0.80 (0.77-0.82)	0.30 (0.20-0.33)	0.94 (0.91-0.94)	3 (2-3)	7 (6-8)	32 (31-33)	2 (2-3)
CSF-only	-	0.86 (0.75-0.96)	0.44 (0.17-0.75)	1.00 (1.00-1.00)	5 (1-9)	6 (2-11)	33 (27-38)	0 (0-0)

Data correspond to the model across 1000 iterations, yielding median accuracy. The 95% confidence interval is shown across 1000 iterations.
FN = false negative; FP = false positive; NPV = negative predictive value; PPV = positive predictive value; TN = true negative; TP = true positive.

Table S6. Model-based risk stratification for LBP_{any}-status

	Whole cohort			Clinical parkinsonism			Clinical AD			Clinically unimpaired		
	**All**	**LBP_{any}-**	**LBP_{any}+**	**All**	**LBP_{any}-**	**LBP_{any}+**	**All**	**LBP_{any}-**	**LBP_{any}+**	**All**	**LBP_{any}-**	**LBP_{any}+**
80% sensitivity												
**Low risk**	152	137 (90.1)	35 (9.9)	39	32 (82.1)	7 (17.9)	41	31 (75.6)	10 (24.4)	37	29 (78.4)	8 (21.6)
**High risk**	186	51 (27.4)	135 (72.6)	111	12 (10.8)	99 (89.2)	56	23 (41.1)	33 (58.9)	7	5 (71.4)	2 (28.6)
85% sensitivity												
**Low risk**	144	120 (83.3)	24 (16.7)	32	29 (90.6)	3 (9.4)	31	23 (74.2)	8 (25.8)	34	28 (82.4)	6 (17.6)
**High risk**	214	68 (31.8)	146 (68.2)	118	15 (12.7)	103 (87.3)	66	31 (47.0)	35 (53.0)	10	6 (60.0)	4 (40.0)
90% sensitivity												
**Low risk**	113	95 (84.1)	18 (15.9)	23	21 (91.3)	2 (8.7)	23	16 (69.6)	6 (30.4)	31	25 (80.6)	6 (19.4)
**High risk**	245	93 (38.0)	152 (62.0)	127	23 (18.1)	105 (81.9)	75	38 (50.7)	37 (49.3)	13	9 (69.2)	4 (30.8)
95% sensitivity												
**Low risk**	52	44 (84.6)	8 (15.4)	7	6 (85.7)	1 (14.3)	11	9 (81.8)	2 (19.2)	17	13 (76.5)	4 (23.5)
**High risk**	306	144 (47.1)	162 (52.9)	143	38 (26.6)	105 (73.4)	86	45 (52.3)	41 (47.7)	27	21 (77.8)	6 (22.2)

Data are presented as n or n (%). The first column indicates the evaluated strategies with different sensitivity-based thresholds for UPSIT-derived risk stratifcation. For each strategy, the total number of individuals in the low- and high-risk groups are shown, followed by numbers of Lewy body pathology negative (LBP-) and LBP+ participants according to *postmortem* neuropathology measures in any brain region (any). The percentage of LBP_{any}-negatives in the low-risk group and the percentage of LBP_{any}-positives in the high-risk group correspond to each evaluated threshold's NPV and PPV, respectively. AD = Alzheimer's disease; LBP = Lewy body pathology.

Table S7. Two-step workflow performance for detecting any LB pathology

Approach	Sensitivity	Median accuracy (CI)	Median PPV (CI)	Median NPV (CI)	Median TP (CI)	Median FP (CI)	Median TN (CI)	Median FN (CI)
Whole cohort								
Two-step	80%	0.86 (0.85-0.87)	0.98 (0.98-0.98)	0.80 (0.79-0.81)	122 (120-125)	2 (2-3)	186 (185-186)	48 (45-50)
Two-step	85%	0.88 (0.87-0.88)	0.98 (0.97-0.99)	0.82 (0.81-0.83)	130 (128-132)	3 (2-4)	185 (184-186)	40 (38-42)
Two-step	90%	0.89 (0.88-0.89)	0.97 (0.96-0.97)	0.84 (0.83-0.84)	134 (132-136)	5 (4-6)	183 (182-184)	36 (34-38)
Two-step	95%	0.90 (0.90-0.91)	0.96 (0.96-0.96)	0.86 (0.85-0.87)	141 (139-142)	6 (6-6)	182 (182-182)	29 (28-31)
UPSIT-only	80%	0.76 (0.75-0.77)	0.73 (0.71-0.74)	0.80 (0.79-0.81)	136 (133-139)	52 (48-55)	136 (133-140)	34 (31-37)
UPSIT-only	85%	0.74 (0.72-0.75)	0.68 (0.66-0.69)	0.82 (0.81-0.84)	145 (142-147)	69 (65-74)	119 (114-123)	25 (23-28)
UPSIT-only	90%	0.69 (0.68-0.71)	0.62 (0.61-0.64)	0.85 (0.83-0.86)	153 (150-155)	92 (87-99)	96 (89-101)	17 (15-20)
UPSIT-only	95%	0.58 (0.56-0.59)	0.53 (0.52-0.54)	0.84 (0.80-0.87)	161 (159-163)	143 (138-148)	45 (40-50)	9 (7-11)
CSF-only	-	0.92 (0.89-0.94)	0.96 (0.93-0.99)	0.88 (0.84-0.92)	146 (128-163)	6 (2-11)	181 (164-200)	24 (15-33)
Clinical parkinsonism								
Two-step	80%	0.91 (0.89-0.92)	1.00 (0.99-1.00)	0.76 (0.74-0.79)	92 (91-94)	0 (0-1)	44 (43-44)	14 (12-15)
Two-step	85%	0.93 (0.92-0.93)	0.99 (0.99-1.00)	0.81 (0.79-0.82)	96 (94-96)	0 (0-1)	43 (43-44)	10 (10-12)
Two-step	90%	0.93 (0.93-0.93)	0.99 (0.99-0.99)	0.81 (0.81-0.81)	96 (96-96)	1 (1-1)	43 (43-43)	10 (10-10)
Two-step	95%	0.93 (0.93-0.93)	0.99 (0.99-0.99)	0.81 (0.81-0.81)	96 (96-96)	1 (1-1)	43 (43-43)	10 (10-10)
UPSIT-only	80%	0.89 (0.87-0.89)	0.91 (0.89-0.91)	0.83 (0.80-0.87)	99 (98-101)	10 (10-12)	34 (32-34)	7 (5-8)
UPSIT-only	85%	0.87 (0.86-0.89)	0.87 (0.85-0.89)	0.90 (0.85-0.91)	103 (101-102)	15 (13-18)	29 (26-31)	3 (3-5)
UPSIT-only	90%	0.83 (0.81-0.85)	0.82 (0.80-0.83)	0.91 (0.87-0.92)	104 (103-104)	23 (21-26)	21 (18-23)	2 (2-3)
UPSIT-only	95%	0.74 (0.73-0.75)	0.73 (0.73-0.75)	0.86 (0.75-0.89)	105 (104-105)	38 (36-38)	6 (6-8)	1 (1-2)
CSF-only	-	0.93 (0.89-0.96)	0.99 (0.97-1.00)	0.82 (0.71-0.90)	96 (84-108)	1 (0-3)	43 (32-54)	10 (5-16)
Clinical Alzheimer's disease								
Two-step	80%	0.85 (0.84-0.85)	0.97 (0.97-0.97)	0.79 (0.78-0.79)	29 (28-29)	1 (1-1)	53 (53-53)	14 (14-15)
Two-step	85%	0.86 (0.85-0.86)	0.97 (0.97-0.97)	0.80 (0.79-0.80)	30 (29-30)	1 (1-1)	53 (53-53)	13 (13-14)
Two-step	90%	0.86 (0.86-0.86)	0.97 (0.97-0.97)	0.80 (0.80-0.80)	30 (30-30)	1 (1-1)	53 (53-53)	13 (13-13)

Two-step	95%	0.89 (0.88-0.89)	0.97 (0.97-0.97)	0.84 (0.83-0.84)	33 (32-33)	1 (1-1)	53 (53-53)	10 (10-11)
UPSIT-only	80%	0.65 (0.62-0.67)	0.58 (0.55-0.60)	0.75 (0.73-0.76)	33 (32-33)	24 (22-26)	30 (28-32)	10 (10-11)
UPSIT-only	85%	0.60 (0.57-0.62)	0.53 (0.51-0.55)	0.73 (0.69-0.77)	34 (33-36)	30 (29-33)	24 (21-25)	9 (7-10)
UPSIT-only	90%	0.55 (0.52-0.58)	0.49 (0.47-0.51)	0.73 (0.68-0.76)	37 (36-37)	38 (35-40)	16 (14-19)	6 (6-7)
UPSIT-only	95%	0.50 (0.47-0.52)	0.47 (0.46-0.48)	0.76 (0.67-0.82)	41 (39-41)	46 (44-48)	8 (6-10)	2 (2-4)
CSF-only	-	0.89 (0.81-0.95)	0.97 (0.91-1.00)	0.84 (0.75-0.92)	33 (24-43)	1 (0-3)	53 (43-63)	10 (5-16)
Clinically unimpaired								
Two-step	80%	0.84 (0.82-0.86)	1.00 (1.00-1.00)	0.83 (0.81-0.85)	3 (2-4)	0 (0-0)	34 (34-34)	7 (6-8)
Two-step	85%	0.86 (0.86-0.86)	1.00 (1.00-1.00)	0.85 (0.85-0.85)	4 (4-4)	0 (0-0)	34 (34-34)	6 (6-6)
Two-step	90%	0.86 (0.84-0.86)	1.00 (0.80-1.00)	0.85 (0.85-0.87)	4 (4-5)	0 (0-1)	34 (33-34)	6 (5-6)
Two-step	95%	0.89 (0.86-0.89)	0.86 (0.83-0.86)	0.89 (0.87-0.89)	6 (5-6)	1 (1-1)	33 (33-33)	4 (4-5)
UPSIT-only	80%	0.73 (0.71-0.77)	0.38 (0.29-0.50)	0.81 (0.78-0.83)	3 (2-4)	5 (3-6)	29 (28-31)	7 (6-8)
UPSIT-only	85%	0.73 (0.71-0.75)	0.40 (0.36-0.44)	0.82 (0.82-0.83)	4 (4-4)	6 (5-7)	28 (27-29)	6 (6-6)
UPSIT-only	90%	0.66 (0.61-0.71)	0.31 (0.27-0.36)	0.81 (0.79-0.83)	4 (4-5)	9 (7-11)	25 (23-27)	6 (5-6)
UPSIT-only	95%	0.43 (0.39-0.48)	0.22 (0.19-0.24)	0.77 (0.71-0.79)	6 (5-6)	21 (19-23)	13 (11-15)	4 (4-5)
CSF-only	-	0.98 (0.93-1.00)	0.92 (0.69-1.00)	1.00 (1.00-1.00)	10 (5-15)	1 (0-3)	33 (27-38)	0 (0-0)

Data correspond to the model across 1000 iterations, yielding median accuracy. The 95% confidence interval is shown across 1000 iterations. FN = false negative; FP = false positive; NPV = negative predictive value; PPV = positive predictive value; TN = true negative; TP = true positive.

Table S8. Model-based risk stratification for brainstem/limbic or neocortical LBP-status

	Whole cohort			Clinical parkinsonism			Clinical AD			Clinically unimpaired		
	**All**	**$LBP_{B/L-N}$-**	**$LBP_{B/L-N}$+**	**All**	**$LBP_{B/L-N}$-**	**$LBP_{B/L-N}$+**	**All**	**$LBP_{B/L-N}$-**	**$LBP_{B/L-N}$+**	**All**	**$LBP_{B/L-N}$-**	**$LBP_{B/L-N}$+**
80% sensitivity												
**Low risk**	227	204 (89.9)	23 (10.1)	65	52 (80.0)	13 (20.0)	58	54 (93.1)	4 (6.9)	42	39 (92.9)	3 (7.1)
**High risk**	131	47 (35.9)	84 (64.1)	85	13 (15.3)	72 (84.7)	39	21 (53.8)	18 (46.2)	2	2 (100.0)	0 (0.0)
85% sensitivity												
**Low risk**	213	197 (93.0)	16 (7.0)	56	50 (89.3)	6 (10.7)	53	49 (92.5)	4 (7.5)	42	39 (92.9)	3 (7.1)
**High risk**	145	54 (37.2)	91 (62.8)	94	15 (16.0)	79 (84.0)	44	26 (59.1)	18 (40.9)	2	2 (100.0)	0 (0.0)
90% sensitivity												
**Low risk**	199	187 (94.0)	12 (6.0)	50	47 (94.0)	3 (6.0)	49	45 (91.8)	4 (8.2)	41	38 (92.7)	3 (7.3)
**High risk**	159	64 (40.3)	95 (59.7)	100	18 (18.0)	82 (82.0)	48	30 (62.5)	18 (37.5)	3	3 (100.0)	0 (0.0)
95% sensitivity												
**Low risk**	171	166 (97.1)	5 (2.9)	40	39 (97.5)	1 (2.5)	40	39 (97.5)	1 (2.5)	37	35 (94.6)	2 (5.4)
**High risk**	187	85 (45.5)	102 (44.5)	110	26 (23.6)	84 (76.4)	57	36 (63.2)	21 (36.8)	7	6 (85.7)	1 (14.3)

Data are presented as n or n (%). The first column indicates the evaluated strategies with different sensitivity-based thresholds for UPSIT-derived risk stratifcation. For each strategy, the total number of individuals in the low- and high-risk groups are shown, followed by numbers of Lewy body pathology negative (LBP-) and LBP+ participants according to *postmortem* neuropathology measures corresponding to Unified Staging System for Lewy Body Disorders (USSLB) stages III. Brainstem/Limbic or IV. Neocortical (B/L-N). The percentage of $LBP_{B/L-N}$-negatives in the low-risk group and the percentage of $LBP_{B/L-N}$-positives in the high-risk group correspond to each evaluated threshold's NPV and PPV, respectively. AD = Alzheimer's disease; B/L-N = brainstem/limbic or neocortical; LBP = Lewy body pathology

Table S9 Two-step workflow performance for detecting brainstem/limbic or neocortical LBP-status

Approach	Sensitivity	Median accuracy (CI)	Median PPV (CI)	Median NPV (CI)	Median TP (CI)	Median FP (CI)	Median TN (CI)	Median FN (CI)
Whole cohort								
Two-step	80%	0.90 (0.89-0.91)	0.86 (0.84-0.88)	0.92 (0.90-0.93)	85 (82-88)	14 (12-16)	237 (235-239)	22 (19-25)
Two-step	85%	0.91 (0.90-0.91)	0.85 (0.83-0.86)	0.93 (0.92-0.94)	90 (87-93)	16 (15-18)	235 (233-236)	17 (14-20)
Two-step	90%	0.91 (0.91-0.92)	0.84 (0.83-0.85)	0.95 (0.94-0.96)	95 (93-97)	19 (17-20)	232 (231-234)	12 (10-14)
Two-step	95%	0.92 (0.91-0.92)	0.81 (0.80-0.82)	0.97 (0.97-0.98)	101 (99-102)	24 (22-26)	227 (225-229)	6 (5-8)
UPSIT-only	80%	0.81 (0.80-0.82)	0.65 (0.63-0.64)	0.90 (0.89-0.91)	85 (82-88)	46 (43-49)	205 (202-208)	22 (19-25)
UPSIT-only	85%	0.80 (0.79-0.82)	0.63 (0.62-0.64)	0.92 (0.91-0.93)	90 (87-93)	53 (50-56)	198 (195-201)	17 (14-20)
UPSIT-only	90%	0.79 (0.78-0.80)	0.60 (0.58-0.62)	0.94 (0.93-0.95)	96 (93-98)	64 (60-68)	187 (183-191)	11 (9-14)
UPSIT-only	95%	0.74 (0.73-(0.75)	0.54 (0.53-0.55)	0.97 (0.96-0.98)	102 (100-103)	87 (82-91)	165 (160-169)	5 (4-7)
CSF-only	-	0.86 (0.83-0.89)	0.69 (0.62-0.76)	0.99 (0.98-1.00)	105 (88-122)	47 (35-59)	204 (186-222)	2 (0-5)
Clinical parkinsonism								
Two-step	80%	0.90 (0.88-0.92)	0.95 (0.95-0.96)	0.85 (0.81-0.88)	74 (71-77)	4 (3-4)	61 (61-62)	11 (8-14)
Two-step	85%	0.93 (0.91-0.94)	0.95 (0.94-0.95)	0.91 (0.87-0.94)	79 (76-81)	4 (4-5)	61 (60-61)	6 (4-9)
Two-step	90%	0.94 (0.93-0.95)	0.93 (0.92-0.94)	0.95 (0.92-0.95)	82 (80-82)	6 (5-7)	59 (58-60)	3 (3-5)
Two-step	95%	0.93 (0.91-0.94)	0.90 (0.88-0.91)	0.97 (0.95-0.98)	83 (82-84)	9 (8-11)	56 (54-57)	2 (1-3)
UPSIT-only	80%	0.84 (0.81-0.86)	0.85 (0.84-0.87)	0.83 (0.79-0.86)	74 (71-77)	13 (11-14)	52 (51-54)	11 (8-14)
UPSIT-only	85%	0.86 (0.84-0.87)	0.84 (0.83-0.85)	0.89 (0.85-0.93)	79 (76-81)	15 (14-16)	50 (49-51)	6 (4-9)
UPSIT-only	90%	0.86 (0.84-0.87)	0.82 (0.80-0.84)	0.94 (0.90-0.96)	82 (80-83)	18 (16-20)	47 (45-49)	3 (2-5)
UPSIT-only	95%	0.83 (0.81-0.84)	0.77 (0.75-0.78)	0.98 (0.95-1.00)	84 (83-85)	26 (4-28)	39 (37-41)	1 (0-2)
CSF-only	-	0.91 (0.86-0.95)	0.87 (0.80-0.93)	0.98 (0.94-1.00)	84 (72-96)	13 (7-19)	52 (41-64)	1 (0-3)
Clinical Alzheimer's disease								
Two-step	80%	0.90 (0.88-0.91)	0.77 (0.73-0.81)	0.93 (0.92-0.95)	17 (16-18)	5 (4-6)	70 (69-71)	5 (4-6)
Two-step	85%	0.89 (0.88-0.91)	0.74 (0.69-0.78)	0.94 (0.93-0.95)	18 (17-18)	6 (5-8)	69 (67-70)	4 (4-5)
Two-step	90%	0.89 (0.88-0.91)	0.70 (0.69-0.74)	0.96 (0.94-0.97)	19 (18-20)	8 (7-8)	67 (67-68)	3 (2-4)

Two-step	95%	0.90 (0.89-0.91)	0.70 (0.69-0.72)	0.99 (0.97-0.99)	21 (20-21)	9 (8-9)	66 (66-67)	1 (1-2)
UPSIT-only	80%	0.74 (0.71-0.76)	0.46 (0.43-0.49)	0.92 (0.90-0.93)	17 (16-18)	20 (18-23)	55 (52-57)	5 (4-6)
UPSIT-only	85%	0.70 (0.68-0.73)	0.42 (0.40-0.45)	0.93 (0.91-0.93)	18 (17-18)	24 (22-27)	51 (48-53)	4 (4-5)
UPSIT-only	90%	0.66 (0.64-0.69)	0.39 (0.37-0.41)	0.94 (0.92-0.96)	19 (18-20)	29 (27-31)	46 (44-48)	3 (2-4)
UPSIT-only	95%	0.61 (0.59-0.63)	0.36 (0.35-0.38)	0.97 (0.95-0.98)	21 (20-21)	37 (34-39)	38 (36-41)	1 (1-2)
CSF-only	-	0.86 (0.78-0.93)	0.61 (0.46-0.79)	0.99 (0.95-1.00)	21 (13-29)	13 (6-19)	62 (52-71)	1 (0-3)
Clinically unimpaired								
Two-step	80%	0.91 (0.91-0.91)	0.00 (0.00-0.00)	0.93 (0.93-0.93)	0 (0-0)	1 (1-1)	40 (40-40)	3 (3-3)
Two-step	85%	0.91 (0.91-0.91)	0.00 (0.00-0.00)	0.93 (0.93-0.93)	0 (0-0)	1 (1-1)	40 (40-40)	3 (3-3)
Two-step	90%	0.91 (0.91-0.91)	0.00 (0.00-0.00)	0.93 (0.93-0.93)	0 (0-0)	1 (1-1)	40 (40-40)	3 (3-3)
Two-step	95%	0.89 (0.89-0.93)	0.00 (0.00-0.50)	0.93 (0.93-0.98)	0 (0-2)	2 (1-2)	39 (39-40)	3 (1-3)
UPSIT-only	80%	0.89 (0.89-0.89)	0.00 (0.00-0.00)	0.93 (0.93-0.93)	0 (0-0)	2 (2-2)	39 (39-39)	3 (3-3)
UPSIT-only	85%	0.89 (0.89-0.89)	0.00 (0.00-0.00)	0.93 (0.93-0.93)	0 (0-0)	2 (2-2)	39 (39-39)	3 (3-3)
UPSIT-only	90%	0.86 (0.84-0.89)	0.00 (0.00-0.00)	0.93 (0.93-0.93)	0 (0-0)	3 (2-4)	38 (37-39)	3 (3-3)
UPSIT-only	95%	0.80 (0.77-0.84)	0.00 (0.00-0.00)	0.92 (0.92-0.97)	0 (0-2)	7 (5-7)	34 (34-36)	3 (1-3)
CSF-only	-	0.82 (0.71-0.93)	0.00 (0.00-1.00)	1.00 (1.00-1.00)	3 (0-7)	8 (3-13)	33 (27-38)	0 (0-0)

Data correspond to the model across 1000 iterations, yielding median accuracy. The 95% confidence interval is shown across 1000 iterations. FN = false negative; FP = false positive; NPV = negative predictive value; PPV = positive predictive value; TN = true negative; TP = true positive.

Table S10. Two-step workflow performance for detecting cortical LB pathology in individuals with UPSIT within 5 years of death

Approach	Sensitivity	Median accuracy (CI)	Median PPV (CI)	Median NPV (CI)	Median TP (CI)	Median FP (CI)	Median TN (CI)	Median FN (CI)
Whole cohort								
Two-step	80%	0.92 (0.90-0.93)	0.95 (0.95-0.98)	0.90 (0.88-0.91)	80 (77-83)	4 (2-4)	188 (188-190)	22 (19-25)
Two-step	85%	0.93 (0.92-0.94)	0.96 (0.95-0.97)	0.92 (0.90-0.93)	85 (82-88)	4 (3-4)	188 188-189)	17 (14-20)
Two-step	90%	0.95 (0.94-0.95)	0.95 (0.94-0.96)	0.94 (0.93-0.95)	91 (88-93)	5 (4-6)	187 (186-188)	11 (9-14)
Two-step	95%	0.94 (0.93-0.95)	0.89 (0.87-0.92)	0.97 (0.96-0.97)	96 (94-97)	12 (9-15)	180 (177-183)	6 (5-8)
UPSIT-only	80%	0.82 (0.81-0.83)	0.72 (0.70-0.73)	0.89 (0.87-0.90)	81 (78-84)	32 (30-35)	160 (157-162)	21 (18-24)
UPSIT-only	85%	0.82 (0.80-0.83)	0.69 (0.67-0.71)	0.91 (0.89-0.92)	86 (83-89)	38 (35-42)	154 (150-157)	16 (13-19)
UPSIT-only	90%	0.79 (0.78-0.80)	0.64 (0.62-0.66)	0.93 (0.92-0.95)	92 (89-94)	52 (48-55)	140 (137-144)	10 (8-13)
UPSIT-only	95%	0.69 (0.65-0.72)	0.53 (0.50-0.56)	0.96 (0.94-0.97)	97 (95-98)	87 (76-99)	105 (93-116)	5 (4-7)
CSF-only	-	0.92 (0.88-0.95)	0.81 (0.73-0.88)	0.99 (0.98-1.00)	101 (85-118)	24 (15-34)	168 (151-185)	1 (0-3)
Clinical parkinsonism								
Two-step	80%	0.90 (0.87-0.92)	1.00 (1.00-1.00)	0.77 (0.73-0.80)	65 (62-67)	0 (0-0)	41 (41-41)	12 (10-15)
Two-step	85%	0.93 (0.91-0.95)	1.00 (1.00-1.00)	0.84 (0.79-0.87)	69 (66-71)	0 (0-0)	41 (41-41)	8 (6-11)
Two-step	90%	0.96 (0.94-0.98)	1.00 (0.99-1.00)	0.89 (0.85-0.93)	72 (70-74)	0 (0-1)	41 (40-41)	5 (3-7)
Two-step	95%	0.96 (0.94-0.98)	0.96 (0.95-0.99)	0.95 (0.93-0.98)	75 (74-76)	3 (1-4)	38 (37-40)	2 (1-3)
UPSIT-only	80%	0.84 (0.81-0.86)	0.89 (0.88-0.91)	0.75 (0.70-0.79)	66 (63-68)	8 (7-9)	33 (32-34)	11 (9-14)
UPSIT-only	85%	0.86 (0.84-0.88)	0.89 (0.86-0.90)	0.82 (0.76-0.87)	70 (67-72)	9 (8-11)	32 (30-33)	7 (5-10)
UPSIT-only	90%	0.86 (0.84-0.87)	0.85 (0.83-0.87)	0.88 (0.83-0.93)	73 (71-75)	13 (11-15)	28 (26-30)	4 (2-6)
UPSIT-only	95%	0.81 (0.77-0.84)	0.78 (0.74-0.81)	0.96 (0.90-1.00)	76 (75-77)	22 (18-27)	19 (14-23)	1 (0-2)
CSF-only	-	0.96 (0.92-0.99)	0.95 (0.90-0.99)	0.97 (0.91-1.00)	76 (66-86)	4 (1-8)	37 (28-47)	1 (0-3)
Clinical Alzheimer's disease								
Two-step	80%	0.93 (0.92-0.95)	0.88 (0.87-0.93)	0.93 (0.93-0.95)	13 (13-14)	2 (1-2)	55 (55-56)	4 (3-4)
Two-step	85%	0.93 (0.92-0.95)	0.88 (0.87-0.93)	0.95 (0.93-0.97)	14 (13-15)	2 (1-2)	55 (55-55)	3 (2-4)
Two-step	90%	0.95 (0.93-0.96)	0.88 (0.83-0.89)	0.98 (0.95-0.98)	16 (14-16)	2 (2-3)	55 (54-55)	1 (1-3)

Two-step	95%	0.93 (0.92-0.95)	0.80 (0.76-0.84)	0.98 (0.98-0.98)	16 (16-16)	4 (3-5)	53 (52-54)	1 (1-1)
UPSIT-only	80%	0.74 (0.72-0.77)	0.46 (0.43-0.50)	0.91 (0.91-0.94)	13 (13-14)	15 (13-18)	42 (39-44)	4 (3-4)
UPSIT-only	85%	0.70 (0.68-0.74)	0.42 (0.39-0.47)	0.93 (0.91-0.95)	14 (13-15)	19 (16-21)	38 (36-41)	3 (2-4)
UPSIT-only	90%	0.68 (0.64-0.70)	0.41 (0.38-0.43)	0.97 (0.92-0.97)	16 (14-16)	23 (21-25)	34 (32-36)	1 (1-3)
UPSIT-only	95%	0.51 (0.45-0.57)	0.31 (0.29-0.34)	0.96 (0.94-0.96)	16 (16-16)	35 (31-40)	22 (17-26)	1 (1-1)
CSF-only	-	0.93 (0.88-0.99)	0.78 (0.59-0.95)	1.00 (1.00-1.00)	17 (10-25)	5 (1-9)	52 (44-59)	0 (0-0)
Clinically unimpaired								
Two-step	80%	0.90 (0.90-0.90)	1.00 (1.00-1.00)	0.90 (0.90-0.90)	1 (1-1)	0 (0-0)	35 (35-35)	4 (4-4)
Two-step	85%	0.90 (0.90-0.90)	1.00 (1.00-1.00)	0.90 (0.90-0.90)	1 (1-1)	0 (0-0)	35 (35-35)	4 (4-4)
Two-step	90%	0.88 (0.88-0.90)	0.50 (0.50-1.00)	0.90 (0.90-0.90)	1 (1-1)	1 (0-1)	34 (34-35)	4 (4-4)
Two-step	95%	0.90 (0.88-0.93)	0.67 (0.50-0.75)	0.94 (0.90-0.94)	3 (1-3)	1 (1-2)	34 (33-34)	2 (2-4)
UPSIT-only	80%	0.90 (0.90-0.90)	1.00 (1.00-1.00)	0.90 (0.90-0.90)	1 (1-1)	0 (0-0)	35 (35-35)	4 (4-4)
UPSIT-only	85%	0.90 (0.85-0.90)	1.00 (0.33-1.00)	0.90 (0.89-0.90)	1 (1-1)	0 (0-2)	35 (33-35)	4 (4-4)
UPSIT-only	90%	0.83 (0.80-0.88)	0.25 (0.20-0.50)	0.89 (0.89-0.90)	1 (1-1)	3 (1-4)	32 (31-34)	4 (4-4)
UPSIT-only	95%	0.78 (0.70-0.83)	0.27 (0.14-0.38)	0.93 (0.88-0.94)	3 (1-3)	7 (5-9)	28 (26-30)	2 (2-4)
CSF-only	-	0.85 (0.75-0.95)	0.46 (0.14-0.78)	1.00 (1.00-1.00)	5 (1-10)	6 (2-10)	29 (23-35)	0 (0-0)

294 individuals were included. Data correspond to the model across 1000 iterations, yielding median accuracy. The 95% confidence interval is shown across 1000 iterations. FN = false negative; FP = false positive; NPV = negative predictive value; PPV = positive predictive value; TN = true negative; TP = true positive.

Table S11. PPMI cohort characteristics at baseline

	**Whole cohort (*N*=1209)**
**Age, years**	65.1 (8.7)
**Sex, n female**	566 (46.8%)
**UPSIT score**	23.9 (8.2)
**CSF SAA+, n**	832 (68.8%)
**Primary clinical diagnosis**	
Idiopathic PD	674 (55.7%)
Alzheimer's disease	2 (0.2%)
Corticobasal syndrome	1 (0.1%)
Dementia with Lewy bodies	5 (0.4%)
Essential tremor	20 (1.7%)
Juvenile autosomal recessive parkinsonsim	1 (0.1%)
Control	470 (38.9%)
Spinocerebellar Ataxia	1 (0.1%)
Other neurological disorder(s)	1 (0.1%)

CSF = cerebrospinal fluid; PD = Parkinson's disease; SAA = Seed Amplification Assay; UPSIT = University of Pennsylvania Smell Identification Test.

Table S12. Non-degenerative confounders of olfactory function in false positives and true positives based on UPSIT risk-stratification

	**False positive (*N*=107)**	**True positive (*N*=131)**	***P* value**
**Smoking** (>0 cigarettes a day)			0.188
No	44 (41.1%)	76 (58.0%)	
Yes	63 (58.9%)	55 (42.0%)	
**Alohol** (>0 units per day)			0.173
No	75 (70.1%)	107 (81.7%)	
Yes	32 (29.9%)	24 (18.3%)	
**History of traumatic brain injury**			0.620
No	83 (77.6%)	104 (79.4%)	
Yes	24 (22.4%)	27 (20.6%)	
**History of sinusitus / rhinitus**			0.176
No	78 (72.9%)	98 (74.8%)	
Yes	29 (27.1%)	33 (25.2%)	

Presence of potential confounders of olfactory impairment. *P* values indicates results from two-sided chi-squared tests.

Figure S1. *Antemortem* UPSIT scores across *postmortem* Lewy body pathology

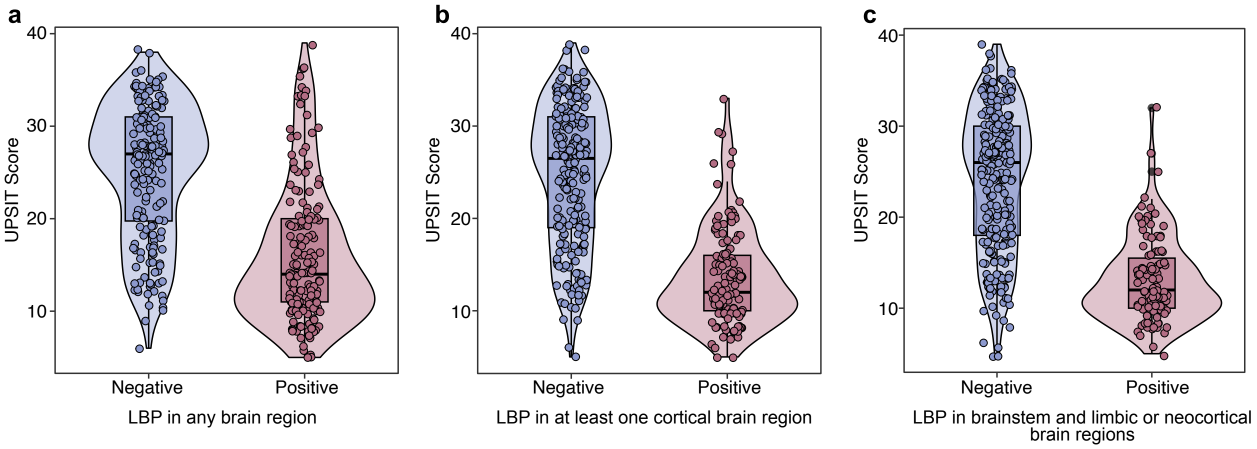

Boxplots showing UPSIT scores for LB-/LB+ groups, measured as (**a**) any brain region showing Lewy body pathology, (**b**) any cortical brain region showing Lewy body pathology, and (**c**) Lewy body pathology corresponding to Unified Staging System for Lewy Body Disorders (USSLB) stages III. Brainstem/Limbic or IV. Neocortical. Boxplots show the median, lower, and upper quartiles with whiskers representing minimum and maximum values. Source data are provided as a Source Data file. LBP = Lewy body pathology; UPSIT = University of Pennsylvania Smell Identification Test.

Figure S2. UPSIT-based risk classification according to lenient and stringent probability thresholds

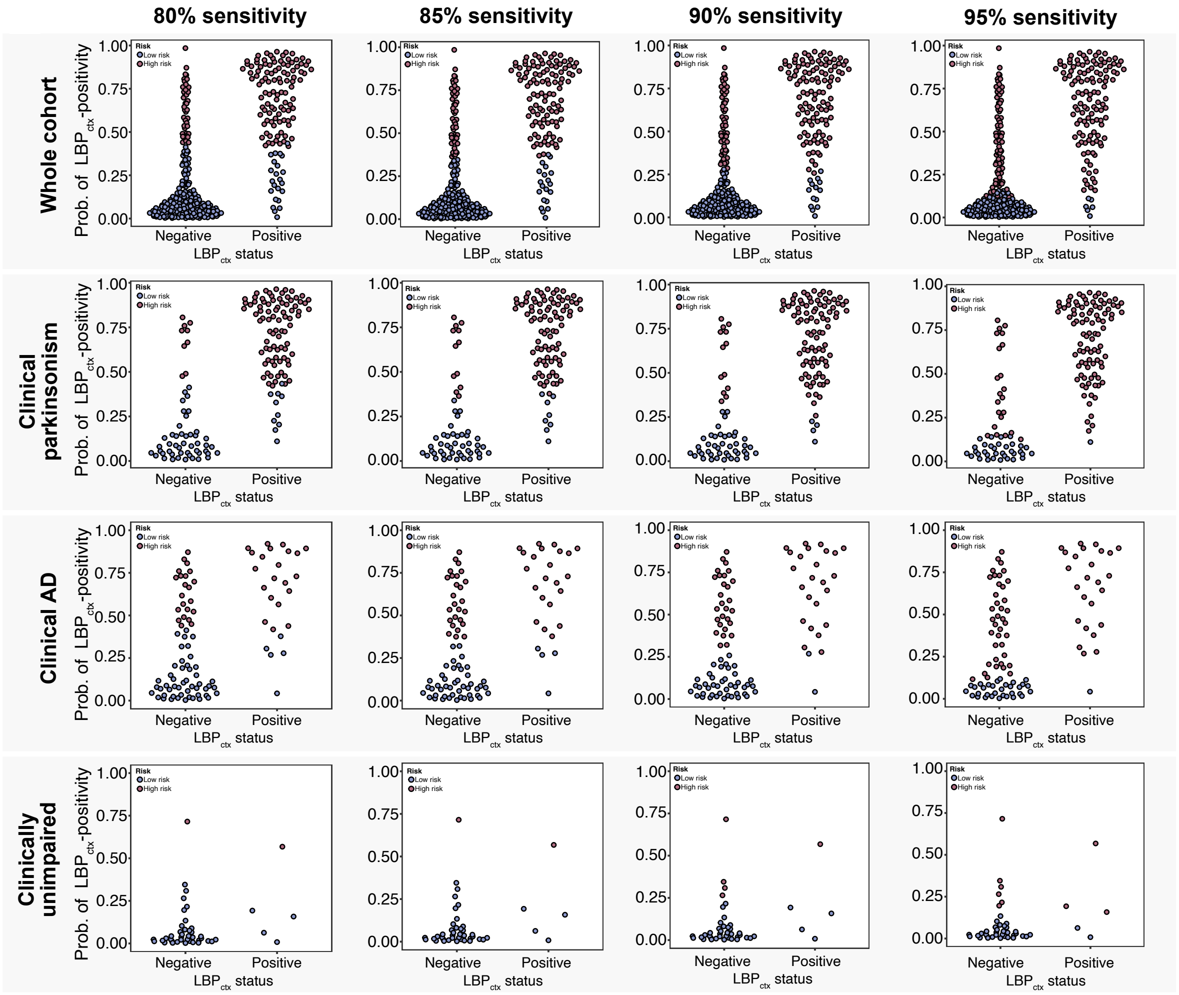

Distribution of predicted probabilities of cortical Lewy body pathology (LBP_{ctx}) positivity based on a logistic regression model including UPSIT scores, age, and sex as predictors. Probability thresholds with 80%, 85%, 90%, and 95% sensitivity were used to classify individuals as low (blue dots) or high (red dots) risk of having cortical LBP. ctx = cortex; LBP = Lewy body pathology; UPSIT = University of Pennsylvania Smell Identification Test.

Figure S3. Results summary against any and brainstem/limbic or neocortical LBP status

a

Patient population	Smell test-based high risk (95% sensitivity)	Lewy body positivity based on CSF SAA in high risk group	Lewy body positivity based on *postmortem* neuropathology in high risk CSF SAA positive group	Reduction in CSF tests
Whole cohort, *n* = 358, 47.5% LBP_{any}+	85.5%	48.0%	95.9%	14.5%
Parkinsonian symptoms, *n* = 150, 70.7% LBP_{any}+	95.3%	67.8%	99.0%	4.7%
Clinical Alzheimer's disease, *n* = 97, 44.3% LBP_{any}+	88.7%	39.5%	97.1%	11.3%
Clinically unimpaired *n* = 44, 22.7% LBP_{any}+	61.4%	25.9%	85.7%	38.6%

b

Patient population	Smell test-based high risk (95% sensitivity)	Lewy body positivity based on CSF SAA in high risk group	Lewy body positivity based on *postmortem* neuropathology in high risk CSF SAA positive group	Reduction in CSF tests
Whole cohort, *n* = 358, 29.9% $LBP_{B/L-N}$+	52.2%	48.0%	66.8%	47.8%
Parkinsonian symptoms, *n* = 150, 56.7% $LBP_{B/L-N}$+	73.3%	67.8%	83.6%	26.7%
Clinical Alzheimer's disease, *n* = 97, 22.7% $LBP_{B/L-N}$+	58.8%	39.5%	52.6%	41.2%
Clinically unimpaired *n* = 44, 6.8% $LBP_{B/L-N}$+	15.9%	25.9%	42.9%	84.1%

Summary of the proportion of individuals selected as high-risk, Lewy body (LB)-positive based on CSF, LB-positive based on *postmortem* neuropathology in (**a**) any region (LBP_{any}+) and (**b**) brainstem and limbic or neocortical LBP stages ($LBP_{B/L-N}$) and the reduction in CSF tests, for each of the four clinical subgroups. B/L-N = brainstem/limbic or neocortical; CSF = cerebrospinal fluid; LBP = Lewy body pathology; SAA = seed amplification assay.
