# Supplementary material for: Two-step detection of Lewy body pathology via smell-function testing and CSF α-synuclein seed amplification
Source: Nat Commun. 2025 Aug 5;16:7182. doi: 10.1038/s41467-025-62458-7 (PMC12325632; doi:10.1038/s41467-025-62458-7)
Supplement: Supplementary file 2 — Reporting Summary [file 41467_2025_62458_MOESM2_ESM.pdf]

nature portfolio

Corresponding author(s): Oskar Hansson
Sophie E. Mastenbroek

Last updated by author(s): Jul 8, 2025

Reporting Summary

Nature Portfolio wishes to improve the reproducibility of the work that we publish. This form provides structure for consistency and transparency in reporting. For further information on Nature Portfolio policies, see our Editorial Policies and the Editorial Policy Checklist.

Statistics

For all statistical analyses, confirm that the following items are present in the figure legend, table legend, main text, or Methods section.

n/a	Confirmed	
[ ]	[x]	The exact sample size (*n*) for each experimental group/condition, given as a discrete number and unit of measurement
[ ]	[x]	A statement on whether measurements were taken from distinct samples or whether the same sample was measured repeatedly
[ ]	[x]	The statistical test(s) used AND whether they are one- or two-sided<br>*Only common tests should be described solely by name; describe more complex techniques in the Methods section.*
[ ]	[x]	A description of all covariates tested
[ ]	[x]	A description of any assumptions or corrections, such as tests of normality and adjustment for multiple comparisons
[ ]	[x]	A full description of the statistical parameters including central tendency (e.g. means) or other basic estimates (e.g. regression coefficient) AND variation (e.g. standard deviation) or associated estimates of uncertainty (e.g. confidence intervals)
[ ]	[x]	For null hypothesis testing, the test statistic (e.g. *F*, *t*, *r*) with confidence intervals, effect sizes, degrees of freedom and *P* value noted<br>*Give P values as exact values whenever suitable.*
[x]	[ ]	For Bayesian analysis, information on the choice of priors and Markov chain Monte Carlo settings
[x]	[ ]	For hierarchical and complex designs, identification of the appropriate level for tests and full reporting of outcomes
[x]	[ ]	Estimates of effect sizes (e.g. Cohen's *d*, Pearson's *r*), indicating how they were calculated

Our web collection on statistics for biologists contains articles on many of the points above.

Software and code

Policy information about availability of computer code

Data collection	No software was used.
Data analysis	Statistical analyses were performed in R version 4.4.2.

For manuscripts utilizing custom algorithms or software that are central to the research but not yet described in published literature, software must be made available to editors and reviewers. We strongly encourage code deposition in a community repository (e.g. GitHub). See the Nature Portfolio guidelines for submitting code & software for further information.

Data

Policy information about availability of data

All manuscripts must include a data availability statement. This statement should provide the following information, where applicable:

- Accession codes, unique identifiers, or web links for publicly available datasets
- A description of any restrictions on data availability
- For clinical datasets or third party data, please ensure that the statement adheres to our policy

Anonymized data from the Arizona Study of Aging and Neurodegenerative Disorders/Brain and Body Donation Program will be shared by request as long as data transfer is in agreement with USA legislation (Privacy Rule of the Health Insurance Portability and Accountability Act). Source data are provided with this paper. Data used in the preparation of this article was obtained on [2025-05-05] from the Parkinson's Progression Markers Initiative (PPMI) database (www.ppmi-info.org/access-data-

specimens/download-data), RRID:SCR_006431. For up-to-date information on the study, visit www.ppmi-info.org. This analysis used DaTscan and αSyn-SAA results for prodromal participants, obtained from PPMI upon request after approval by the PPMI Data Access Committee.

Research involving human participants, their data, or biological material

Policy information about studies with human participants or human data. See also policy information about sex, gender (identity/presentation), and sexual orientation and race, ethnicity and racism.

Reporting on sex and gender	We used the term "sex" throughout the manuscript. Sex was determined based on self-reporting. Statistical analyses included sex as a predictor.
Reporting on race, ethnicity, or other socially relevant groupings	This study did not include categorization of race, ethnicity and/or other socially relevant groupings.
Population characteristics	Detailed information is given in Supplementary Tables 1 and 10. In short, we present results for primary analyses from the Arizona Study of Aging and Neurodegenerative Disorders (AZSAND)/Brain and Body Donation Program BBDP). 358 neuropathological samples with antemortem UPSIT scores and postmortem ventricular brain CSF were included. Mean age at death was 86.2±7.8 years. The cohort consisted of healthy controls and individuals with neurodegenerative diseases.<br><br>In addition, we present results for secondary analyses from the Parkinson's Progression Markers Initiative (PPMI) cohort.1209 individuals with in vivo CSF α-syn SAA and UPSIT available were included. Mean age was 65.1±8.7 years and 46.8% was female.
Recruitment	The Arizona Study of Aging and Neurodegenerative Disorders (AZSAND)/Brain and Body Donation Program (BBDP) is part of the Banner Sun Health Research Institute. AZSAND/BBDP recruitment is directed at cognitively normal elderly and subjects with a clinical diagnosis of Alzheimer's disease (AD), PD, or cancer. The normal subjects are volunteers that are recruited from and reside in the surrounding community while the AD and PD subjects are recruited both from the community and from neurologists' offices.<br><br>The Parkinson's Progression Markers Initiative (PPMI) primarily recruits healthy controls, prodromal synucleinopathies, and PD patients. Recruitment is performed in close collaboration with the Michael J Fox Foundation. Recruitment is performed through international publicity campains and referrals by physicians.
Ethics oversight	All subjects signed written informed consent approved by either the Banner Sun Health Institutional Review Board or the AZSAND/BBDP: Western IRB (Seattle, WA). In the United States, as in much of the world, all medical research on human subjects is ethically and legally guided by the Declaration of Helsinki (http://www.wma.net/en/30publications/10policies/b3/). Other documents of historical importance include the Nuremburg Code and Declaration of Geneva. The principles of the Declaration of Helsinki are honored in the US legal system within the Code of Federal Regulations (CFR),11 where they are summarized as the Common Rule. The Common Rule is administered by the US Office of Human Research Protection, which exists to ensure that all human subject research will adhere to the written standards. The health information of human subjects is protected by the Health Insurance Portability and Accountability Act of 1996 (HIPAA).<br><br>PPMI: The study was approved by the institutional review board at each site, and participants provided written informed consent.

Note that full information on the approval of the study protocol must also be provided in the manuscript.

Field-specific reporting

Please select the one below that is the best fit for your research. If you are not sure, read the appropriate sections before making your selection.

- [x] Life sciences
- [ ] Behavioural & social sciences
- [ ] Ecological, evolutionary & environmental sciences

For a reference copy of the document with all sections, see nature.com/documents/nr-reporting-summary-flat.pdf

Life sciences study design

All studies must disclose on these points even when the disclosure is negative.

Sample size	The study included a large sample size (N=358 neuropathological samples). There is no indication that we were insufficiently powered for these analyses.
Data exclusions	The data were limited to the subset of the source cohort that had antemortem UPSIT scores and postmortem ventricular brain CSF.
Replication	To ensure replicability/generalizability of the reported two-step workflow performance, we performed 5-fold cross-validation, with a different training and test dataset for each fold. In addition, we repeated all analyses across 1000 iterations.<br>In addition, we validated the two-step workflow in a second, in vivo cohort, with 1209 subjects.
Randomization	In this study (observational) no allocation into experimental groups were performed, therefore randomization is not relevant to this study. Age and sex were included in the statistical logistic regression models.

Blinding	Postmortem microscopic examinations were performed blinded to clinical diagnosis by a single neuropathologist (T.G.B.).

Reporting for specific materials, systems and methods

We require information from authors about some types of materials, experimental systems and methods used in many studies. Here, indicate whether each material, system or method listed is relevant to your study. If you are not sure if a list item applies to your research, read the appropriate section before selecting a response.

Materials & experimental systems

n/a	Involved in the study
☒	☐ Antibodies
☒	☐ Eukaryotic cell lines
☒	☐ Palaeontology and archaeology
☒	☐ Animals and other organisms
☒	☐ Clinical data
☒	☐ Dual use research of concern
☒	☐ Plants

Methods

n/a	Involved in the study
☒	☐ ChIP-seq
☒	☐ Flow cytometry
☒	☐ MRI-based neuroimaging

Plants

Seed stocks	*Report on the source of all seed stocks or other plant material used. If applicable, state the seed stock centre and catalogue number. If plant specimens were collected from the field, describe the collection location, date and sampling procedures.*
Novel plant genotypes	*Describe the methods by which all novel plant genotypes were produced. This includes those generated by transgenic approaches, gene editing, chemical/radiation-based mutagenesis and hybridization. For transgenic lines, describe the transformation method, the number of independent lines analyzed and the generation upon which experiments were performed. For gene-edited lines, describe the editor used, the endogenous sequence targeted for editing, the targeting guide RNA sequence (if applicable) and how the editor was applied.*
Authentication	*Describe any authentication procedures for each seed stock used or novel genotype generated. Describe any experiments used to assess the effect of a mutation and, where applicable, how potential secondary effects (e.g. second site T-DNA insertions, mosiacism, off-target gene editing) were examined.*
